# Evaluation of anti-atherosclerotic effects of Sitagliptin via modulation of the mTOR pathway in male rabbits

**DOI:** 10.25122/jml-2022-0298

**Published:** 2023-03

**Authors:** Hussam Hamza Sahib, Bassim Mohammad, Najah Rayish Hadi

**Affiliations:** 1 Department of Clinical Laboratory Sciences, College of Pharmacy, University of Al-Qadisiyah, Diwaniya, Iraq; 2 Department of Pharmacology and Therapeutics, College of Medicine, University of Al-Qadisiyah, Diwaniya, Iraq; 3 Department of Pharmacology & Therapeutics, Faculty of Medicine, University of Kufa, Kufa, Iraq

**Keywords:** anti-atherosclerotic effects, Sitagliptin, modulation of mTOR pathway

## Abstract

Atherosclerosis is a common and serious vascular disease that underlies many cardiovascular and cerebrovascular illnesses, including heart attack and stroke. Atherosclerosis-related illnesses have increased in prevalence and now pose a substantial burden on individuals and society. Autophagy (AP) is a process in which cytoplasmic components are engulfed by a double-membrane structure, such as defective organelles and aged, damaged, and flawed proteins. Autophagy is essential for maintaining a proper cellular equilibrium and plays a vital homeostatic role in physiological settings by liberating nutrients from macromolecules and removing undesirable cellular components. This study aimed to investigate the effect of Sitagliptin on the progression of atherosclerosis. Twenty-one male New Zealand White rabbits weighing 2-2.5 kg each were split into three groups: normal control, atherogenic control, and Sitagliptin-treated. The following parameters: serum triglycerides (TG), total cholesterol (TC), LDL, and a tissue autophagy marker (p62) using ELISA, aortic mRNA expression of mTORC1 marker using Real-Time Quantitative PCR(RT-qPCR), and histological inspection of the aorta were assessed. The mRNA expression of mTORC1 and the lipid profile of aortic tissue are considerably elevated in atherogenic diet-fed animals. Histopathological analysis confirmed the presence of a substantial atherosclerotic lesion in the animals fed an atherogenic diet. However, compared to an atherogenic control group, Sitagliptin dramatically reduced lipid profile, P62 aortic level, and mRNA expression of mTORC1. Sitagliptin medication slowed the development of atherosclerosis via increasing autophagy through suppression of the mTORC1 signaling pathway.

## INTRODUCTION

Atherosclerosis is a systemic vascular disease that is a major cause of cardiovascular and cerebrovascular illnesses [1]. The prevalence of disorders associated with atherosclerosis has increased, making it a leading cause of mortality worldwide and a significant burden on individuals and society [2]. The carotid and coronary arteries are especially at risk, although other arteries may also be impacted [3]. Inflammation plays a crucial role in the development of the disease, contributing to well-known risk factors and being an independent cause of atherosclerotic plaque progression. The hallmark of atherosclerosis is inflammation, which is present from the early stages of the disease due to lipoprotein storage and immune cell infiltration. The inflammatory processes continue through the development of fragile plaques prone to rupture [4]. Autophagy (AP) is a cellular process in which cytoplasmic components are engulfed by a double-membrane structure, such as defective organelles, aged and damaged proteins, and flawed proteins during biosynthesis, on their way to destruction in lysosomes. This process is crucial to maintain cellular homeostasis and serves as an important homeostatic function in releasing nutrients from macromolecules and degrading unwanted cellular components [5,6]. Lesional macrophages undergo autophagy, which reduces apoptosis and improper efferocytosis. However, impaired autophagy has been linked to increased oxidative stress and necrosis in advanced atherosclerotic plaques [7]. By shielding cells and decreasing the release of inflammatory chemicals, the stimulation of autophagy aids in the stability of atherosclerotic plaques. Autophagy may also restrict apoptosis by inhibiting inflammasome formation and activation [8]. Macrophage autophagy plays a critical role in foam cell production by reducing macrophage oxLDL absorption, increasing macrophage efferocytosis, and promoting cholesterol export. This, in turn, mitigates the severity of AS by decreasing lesional plaque apoptosis and inflammation [9]. Autophagy regulation is a complex process involving several signaling pathways. Some of these pathways rely on mTOR for their function, while others do not. Both the AMPK and mTOR pathways may stimulate autophagy, although the former is more important [10]. As a sensor of cellular energy and nutrients, mTOR is also an important regulator of autophagy. To prevent autophagosome formation, mTOR integrates upstream signals and inhibits ATG1 (autophagy-related gene-1) [11].

When there are enough resources to support growth, mTORC1 can suppress autophagy by integrating upstream signals via the Akt pathways. However, a variety of stimuli, such as hunger and inflammatory oxidative stress, may activate class III P3K-Becln1 which can promote the formation of autophagy-related complexes, including ATG12, ATG5, and ATG16L, as well as ATG8/LC3. On the other hand, the complexes Belin1 and Bl-2 can inactivate class III P3K-Becln1 [12]. Incretin hormones promote insulin secretion, and a class of drugs known as Gliptins, which includes sitagliptin, enhance these physiological activities and are used as an oral anti-diabetic treatment [13]. Sitagliptin, a selective dipeptidyl peptidase-4 (DPP-4) inhibitor, has shown significant success as a monotherapy or in combination with metformin for managing type 2 diabetes by blocking the breakdown of GLP-1 and glucose-dependent insulinotropic polypeptide, which increases their beneficial effects [14]. In addition to managing diabetes, incretin enhancers such as sitagliptin may have positive effects on diabetes pathophysiology and prevent major consequences like atherosclerosis [15]. Recent research by Wenbin et al. shows that sitagliptin can decrease hepatic steatosis by increasing autophagy in ob/ob mice [16].

## MATERIAL AND METHODS

### Animals

Twenty-one adult male New Zealand White rabbits, weighing an average of 2.5 kilograms, were utilized in the experiment. The research was conducted at the Department of Pharmacology, College of Medicine at Al Qadisiyah University, following the institutional policy for Ethics in Animal Research. The rabbits were housed in a controlled environment with a constant humidity of 50% and a 12-hour light/dark cycle. The rabbits were allowed to adapt to their surroundings for two weeks prior to the commencement of the experiment. During this acclimation period, they were given a standard laboratory diet and continuous access to water.

### Treatment

A dosage of 12 mg/kg/day of Sitagliptin (manufactured in China by Hangzhou Hyper Chemicals Limited; Batch number 20112301) dissolved in distilled water (Hangzhou Hyper Chemicals Limited, China; Batch No. 20112301) was utilized [17]. The medication was administered once daily via a stomach tube at a dosage determined based on the weight of the rabbit.

### Atherosclerosis induction

For 8 weeks, rabbits were fed an atherogenic diet (a 2% cholesterol-enriched diet created by adding cholesterol powder to chow pellets), which induced hyperlipidemia and led to the development of atherosclerosis [18].

### Sample collection and processing

Following a 2-week acclimatization period, the rabbits were randomly assigned into three groups of 7 rabbits each: a normal diet control group (NC, Group I), an atherogenic control group fed a high-cholesterol diet (AC, Group II), and a high-cholesterol diet with sitagliptin group (Group III). The NC group received normal rabbit chow, whereas the high-cholesterol diet groups were fed a 2% high-cholesterol (atherogenic) diet. The duration of the experiment was 8 weeks. At the end of the experiment, food was withheld for 16-18 hours, and animals were anesthetized with ketamine (HIKMA pharmaceuticals B.N 3310) at 66mg/kg and xylazine (Alfasan B.N 1004111-07) at 6mg/kg via intramuscular injection [18]. A thoracotomy was performed to open the chest, collect blood directly from the heart, and separate the aorta. Subsequently, the following investigations were performed:

1. Analysis of lipid profile;
2. Macrophage autophagy marker (P62) analysis;
3. qRT-PCR analysis of mTOR expression;
4. Atherosclerosis Histopathological Analysis.

### Tissue sample preparation

The aortic arch was dissected, exteriorized, and freed from connective tissue and fat adhesions. The tissue was then divided into three sections. For 24 hours, the initial tissue sample was immediately immersed in a 10% formaldehyde solution to measure P62 using the ELISA technique. Next, measurement of mTOR mRNA expression by Real-Time Quantitative PCR was performed on the third tissue component (RT-qPCR).

### Extraction of total RNA from the aortic tissue and PCR-based reverse transcription

#### Aortic total RNA extraction

To extract total RNA from aorta samples, a TRIzol® reagent kit was used as recommended by the manufacturer. Homogenization was achieved by adding 750 µl of TRIzol® reagent to a 1-100 mg sample of cardiac tissue. The tubes were filled with 200 µl of chloroform each and were shaken for 15 seconds. The resulting mixture was incubated on ice for five minutes, then centrifuged for 15 minutes at 4C and 12000 rpm. The supernatant was transferred to a new Eppendorf tube, and 500 µl of isopropanol was added. After inverting the tube four to five times, it was incubated at 4C° for 10 minutes and then centrifuged at 4 degrees Celsius at 12000 rpm for 10 minutes. The supernatant was removed, and 1 ml of 80% ethanol was added to the pellet and mixed by vortexing. After centrifugation at 4 degrees Celsius and 12000 rpm for 5 minutes, the RNA pellet was air-dried after the supernatant was removed and dissolved in 70 to 100 µl of free nuclease water. The isolated RNA was stored at -20 degrees Celsius. The primers for the target gene (mTORC1) and housekeeping gene (GAPDH) were designed using the NCBI-Gene Bank database and Primer design online, provided by (Bioneer Company, Korea). Details of the primers are shown in Table 1.

**Table 1:** qPCR primer sequences used.

Primer	Sequence (5'-3')		Product Size	Genbank
**mTORC1**	F	GAATTTGCAGGCGCTGTTTG	117bp	XM_008251668.2
	R	AGGAAAGGCATGACAAAGGC		
**GAPDH**	F	GTCAAGGCTGAGAACGGGAA	95bp	NM_001082253.1
	R	CCAGCATCACCCCACTTGAT		

#### Quantitative Real-Time PCR (qPCR)

Different groups of rabbits were analyzed for their target gene (mTORC1) expression using quantitative Real-Time PCR. The data were normalized by the expression of the housekeeping gene (GAPDH). The GoTaq® qPCR Master Mix kit was used to generate the qPCR master mix for Real-Time PCR, amplifying the GAPDH gene and detecting the target. After adding the resulting mixture to qPCR plate strip tubes and performing Exispin vortex centrifuge mixing for 3 minutes, it was transferred to a Miniopticon Real-Time PCR system for analysis. Using the relative measurement of gene expression levels (fold change) (The CT Method Employing a Reference Gene), the qRT-PCR data for both target and housekeeping genes were evaluated by [19] using the following equation:

∆CT (Test) = CT (target gene, test) – CT (HKG gene, test)

∆CT (Control) =

= CT (target gene, control) – CT (HKG gene, control)

∆∆CT= ∆CT (Test) – ∆CT (Control)

Fold change (target/HKG) = 2 ^- ∆∆CT^

### Histopathological analysis of the aorta

The tissue samples were processed for histological analysis of atherosclerosis according to standard protocol. They were embedded in paraffin and sectioned to a thickness of 5µm. Hematoxylin and eosin were used to stain the tissue sections. Atherosclerotic alterations were assessed using a light microscope at magnifications of 40X and 100X following the categorization of atherosclerosis established by the American Heart Association. This included Type I (initial lesions), Type II (fatty streak lesions), Type III (intermediate lesions), Type IV (atheroma), Type V (advanced lesion), and Type VI (end-stage lesion) (complicated lesion) [20].

### Statistical analysis

Statistical analyses were conducted using IBM SPSS Statistics version 27.0. The data were presented as mean ± standard error of the mean (SEM). Paired t-test was used to compare means within each group over time, while a one-way analysis of variance (ANOVA) was used for comparisons among multiple groups. The histological classification was evaluated using the Mann-Whitney U test. A statistical significance level of P < 0.05 was considered for all tests.

## RESULTS

### Effect of Sitagliptin on serum lipid profile

**Figure 1:**
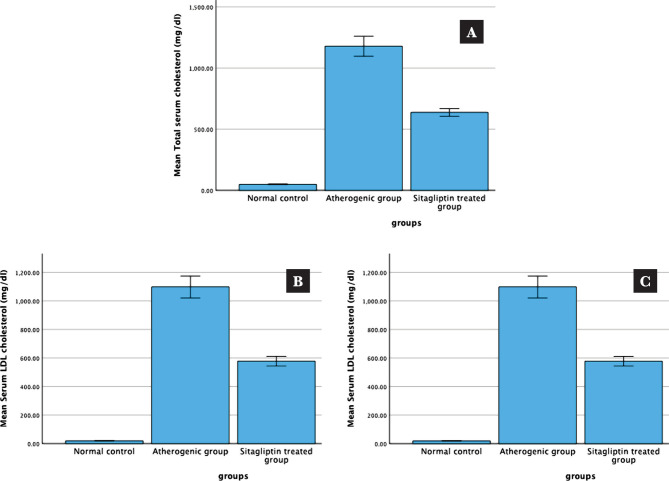
A – Mean changes of total serum cholesterol among the 3 study groups; B – Mean changes of serum triglycerides (TC) (mg/dl) among the study groups; C – Mean changes of serum LDL cholesterol (mg/dl) among the study groups.

All animals given high-cholesterol diets experienced a pronounced change in their lipid profiles, while the sitagliptin group had significantly lower serum lipids than the control group (Figure 1 A–C and Table 2).

**Table 2:** Serum TC, TG, and LDL levels differed across the three groups.

Groups	TC (mg/dl)	TG (mg/dl)	LDL (mg/dl)
**Normal control group**	48.4±0.88	60.1±0.77	19.16±0.715
**Induced untreated group**	1177±33.4*	302.4±20.40*	1097.85±31.25*
**Sitagliptin treated group**	636.8±12.9**	146±12.52**	577.28±13.55**

Results are expressed as Mean±SEM using the paired T-test (N=7 in each group). * – P<0.05, as compared to the NC group; ** – P<0.05, as compared to the AC group.

### Effect of Sitagliptin on mRNA expression levels of mTORC1 in aortic tissues

**Figure 2:**
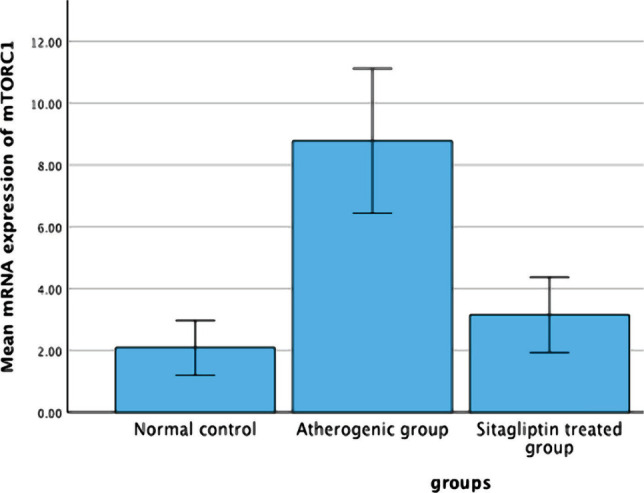
Mean changes in the mRNA expression level of mTORC1 among the study groups.

The aortic mTORC1 mRNA expression was higher in rabbits with atherosclerosis than in the normal control group (P<0.05). However, the sitagliptin-treated group had significantly lower (P<0.05) mTORC1 mRNA expression levels compared to the untreated atherosclerosis group (Figure 2 and Table 3).

**Table 3:** Variations in mTORC1 mRNA expression in rabbits across the three groups.

Groups	mTORC1 mRNA expression
**Normal control group**	2.08±0.361
**Induced untreated group**	8.77±0.955*
**Sitagliptin treated group**	3.14±0.497**

Results are expressed as Mean±SEM using a paired T-test (N=7 in each group). * – P<0.05, as compared to the NC group; ** – P<0.05, as compared to the AC group.

### Effect of Sitagliptin on autophagy marker level (P62)

**Figure 3:**
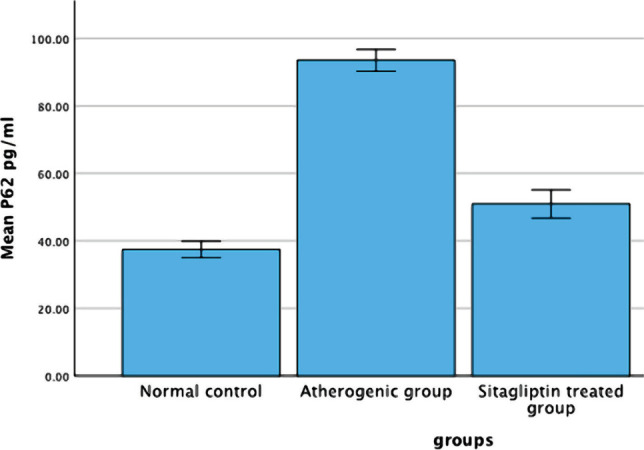
Mean changes of P62 among the study groups.

Significant changes (P<0.05) were observed in P62 levels after 8 weeks of the high cholesterol diet, with a significant difference between the NC and atherogenic groups. Furthermore, there was a significant difference in P62 levels between the atherogenic and the sitagliptin-treated group (Figure 3 and Table 4).

**Table 4:** The average values of the autophagy marker P62 across the three experimental groups.

Groups	Aortic p62 level (pg/ml)
**Normal control group**	37.45±1.01
**Induced untreated group**	93.57±1.32*
**Sitagliptin treated group**	50.96±1.72**

Results are expressed as Mean±SEM using a Student's t-Test (N=7 in each group). * – P<0.05, as compared to the NC group; ** – P<0.05, as compared to the AC group.

### Effect of Sitagliptin on aortic lesion formation induced by an atherogenic diet

**Figure 4:**
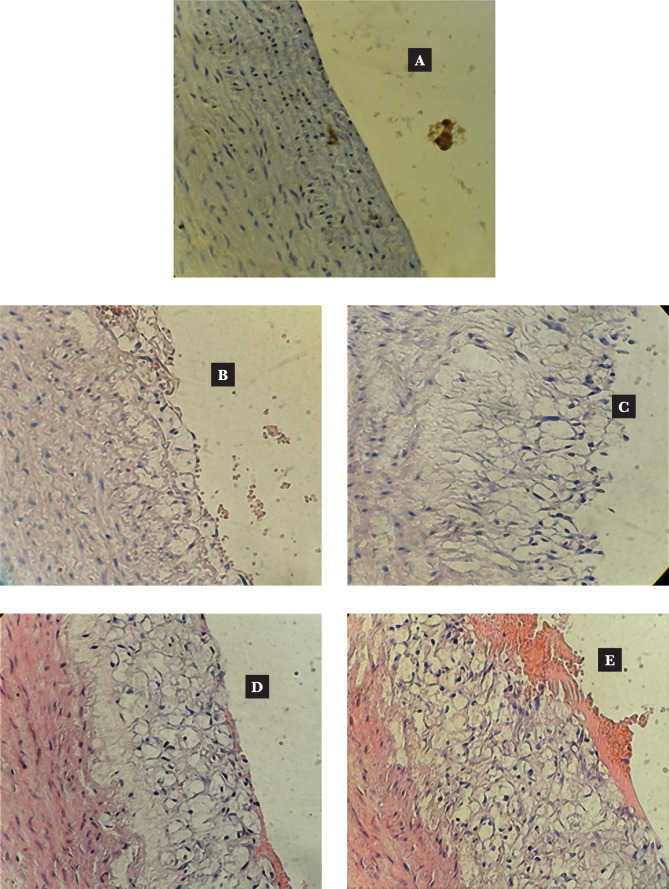
A cross-section of the aortic arch from a hypercholesterolemic rabbit represented atherosclerosis progression (A–E = 100X). A – Normal arterial appearance; B – Initial atherosclerotic lesion characterized by lipid-laden macrophage (foam cells); C – Intermediate atherosclerotic lesion characterized by extracellular lipid pool; D – Advanced atherosclerotic lesion characterized by a core of extracellular lipid; E – Complicated atherosclerotic lesion characterized by hemorrhagic thrombus.

Atherosclerotic aortic arch lesions were categorized as either normal, intermediate, advanced, or complex (Figure 4 A–E). There were significant differences in the median histopathological grade of atherosclerotic changes among the three groups. The atherogenic control (advanced) group had the highest median grade compared to any other group, while the normal diet group had the lowest median (no abnormality). The sitagliptin group had a considerably lower median aortic change (initial) compared to the atherogenic control group (Figure 4).

## DISCUSSION

In this study, we observed that a high-atherogenic diet significantly increased lipid parameters, including TC, TG, and LDL, compared to the control group. These findings agree with a previous study by Najah et al. [21]. Treatment with Sitagliptin significantly reduced the levels of TC, TG, and LDL compared to the atherogenic group, consistent with the results of Sahar et al. [15].

Our results also demonstrated that mTORC1 mRNA expression levels were significantly decreased in the sitagliptin-treated group compared to the atherogenic group. This suggests that Sitagliptin negatively regulates the mTORC1 signaling pathway, consistent with the results of a previous study [22].

We found a statistically significant increase in the P62 level in the atherogenic group compared to the normal control group, comparable to another study [23]. The clearance of intracellular excess or faulty protein by autophagy, a widely conserved mechanism, has regulatory effects on the progression of atherosclerosis [8]. Bioactive molecule p62 is involved in autophagy. The p62 was also involved in identifying and conveying rejected cargos to the autophagosome, which was destroyed along with cargos when autophagy flow was inhibited. However, high levels of p62 can impair the autophagic process. Dysregulation of autophagy has been linked to several diseases, including atherosclerosis [24]. Although several autophagy-stimulating factors are present within the atherosclerotic plaque, such as reactive oxygen species, oxidized lipids, and cytokines, the autophagy process becomes dysfunctional during the development of atherosclerosis [25]. Therefore, autophagy dysfunction causes an increase in p62 levels in the aorta tissue of hypocholesterolemic rabbits. Sitagliptin treatment decreased aortic p62 protein levels in the atherogenic group, suggesting that it restored defective autophagy. These findings suggest that Sitagliptin may promote autophagy.

## CONCLUSION

The study concluded that the Sitagliptin-treated group showed significantly less severe (initial) atherosclerotic lesions compared to the atherogenic group. These findings suggest that Sitagliptin may have therapeutic potential in reducing atherosclerosis. It is hypothesized that this effect may be due to the induction of autophagy, which could be mediated by inhibiting the mTORC1 pathway.

## Personal thanks

We would like to express our gratitude to the members of the Pharmacology and Therapeutic Department of Kufa Medical College for their valuable contributions to this study.
